# Clinical, epidemiological, and spatial characteristics of *Vibrio parahaemolyticus* diarrhea and cholera in the urban slums of Kolkata, India

**DOI:** 10.1186/1471-2458-12-830

**Published:** 2012-09-28

**Authors:** Suman Kanungo, Dipika Sur, Mohammad Ali, Young Ae You, Debottam Pal, Byomkesh Manna, Swapan K Niyogi, Banwarilal Sarkar, Sujit K Bhattacharya, John D Clemens, G Balakrish Nair

**Affiliations:** 1 National Institute of Cholera and Enteric Diseases, Kolkata, India; 2 International Vaccine Institute, Seoul, Republic of Korea; 3 Society for Applied Studies, Salt Lake City, Kolkata, India; 4 UCLA School of Public Health, Los Angeles, USA

**Keywords:** *Vibrio parahaemolyticus*, *Vibrio cholerae*, Cholera, Kolkata

## Abstract

**Background:**

There is not much information on the differences in clinical, epidemiological and spatial characteristics of diarrhea due to *V. cholerae* and *V. parahaemolyticus* from non-coastal areas. We investigated the differences in clinical, epidemiological and spatial characteristics of the two *Vibrio* species in the urban slums of Kolkata, India.

**Methods:**

The data of a cluster randomized cholera vaccine trial were used. We restricted the analysis to clusters assigned to placebo. Survival analysis of the time to the first episode was used to analyze risk factors for *V. parahaemolyticus* diarrhea or cholera. A spatial scan test was used to identify high risk areas for cholera and for *V. parahaemolyticus* diarrhea.

**Results:**

In total, 54,519 people from the placebo clusters were assembled. The incidence of cholera (1.30/1000/year) was significantly higher than that of *V. parahaemolyticus* diarrhea (0.63/1000/year). Cholera incidence was inversely related to age, whereas the risk of *V. parahaemolyticus* diarrhea was age-independent. The seasonality of diarrhea due to the two *Vibrio* species was similar. Cholera was distinguished by a higher frequency of severe dehydration, and *V. parahaemolyticus* diarrhea was by abdominal pain. Hindus and those who live in household not using boiled or treated water were more likely to have *V. parahaemolyticus* diarrhea. Young age, low socioeconomic status, and living closer to a project healthcare facility were associated with an increased risk for cholera. The high risk area for cholera differed from the high risk area for *V. parahaemolyticus* diarrhea.

**Conclusion:**

We report coexistence of the two vibrios in the slums of Kolkata. The two etiologies of diarrhea had a similar seasonality but had distinguishing clinical features. The risk factors and the high risk areas for the two diseases differ from one another suggesting different modes of transmission of these two pathogens.

## Background

*Vibrio parahaemolyticus* diarrhea and cholera diarrhea (due to *Vibrio cholerae* O1 and less commonly *V. cholerae* O139) are both public health concerns. *V. parahaemolyticus* is a halophilic etiologic agent of diarrheal disease, having an ability to produce outbreaks of gastroenteritis [1]. Recently, *V. parahaemolyticus* of specific serotypes were associated with the outbreaks in several parts of the world with the earliest cases being reported from Kolkata, India in 1996 [2,3]. The bacterium was first identified as a cause of seafood-borne illness in Japan in 1950, when 272 individuals became ill and 20 died after the consumption of semidried juvenile sardines [4]. It is associated with three major syndromes of clinical illness: gastroenteritis, wound infections, and septicemia. The most common syndrome is gastroenteritis; the symptoms include diarrhea with abdominal cramps, nausea, vomiting, headache, and low-grade fever [5]. Between 1988 and 1997, a review of infections found that 88% of patients with *V parahaemolyticus* gastroenteritis and 91% of patients with *V. parahaemolyticus* primary septicemia had a known food history reported eating raw oysters [6]. Thus, consumption of crustacean and molluscan shellfish has commonly been implicated in the transmission of *V. parahaemolyticus*.

Cholera infection is often mild or without symptoms, assuming 90% of the infections are asymptomatic [7], but sometimes it can be severe. Approximately one in 20 infected persons has severe disease characterized by profuse watery diarrhea, vomiting, and leg cramps. In these persons, rapid loss of body fluids leads to dehydration, acute renal failure and shock. Without treatment, death can occur within hours. The epidemiological patterns of cholera depend largely on environmental factors including sanitary conditions and social aspects, prior immune status of the population at risk, and the inherent properties of the vibrios themselves. Until the mid-1980s, humans were thought to be the only reservoir of *V. cholerae*. It is now believed that the organism has a free-living cycle and is a natural resident of aquatic environs [8]. A significant marine reservoir of *V. cholerae* is plankton, and the bacterium attaches primarily to zooplankton, specifically copepods [9]. It is now believed that the transmission of the vibrios might occur through water without fecal contamination, and the evidences suggest natural reservoir of two vibrios is the aquatic environment.

Although the reservoir of both the organisms (*V. parahaemolyticus* and *V. cholerae*) is believed to be marine environment, they have the ability to infect people in areas far from coastal areas. However, there is not much information on the differences in clinical, epidemiological and spatial characteristics of diarrhea due to *V. cholerae* and *V. parahaemolyticus* from non-coastal areas. This paper describes these characteristics in the urban slums of, a non-coastal area, Kolkata, India, where a cohort of population was under uniform surveillance for diarrhea.

## Methods

### The study area and data

The study was conducted in urban slum communities in Kolkata, the capital of the state of West Bengal. Kolkata, the third largest city in India, has 14 million inhabitants living within an area of 1,450 km^2^, making it one of the world’s most densely populated cities. The Kolkata Municipal Corporation consists of 141 civic administrative units called wards, with each ward having an office responsible for public health supervised by a medical officer. The study site comprises three contiguous wards (29, 30, and 33) with about 100,000 residents. These residents live in homes tightly-spaced together along winding sewage-littered pathways, and they rely on shared toilets and drinking-water [10]. The area has a high population density. Through the years, extensive subletting has resulted in overcrowding as more and more people are squeezed into available housing [11]. Sufficient water supply and sanitary facilities are unavailable in the area. Several households share one or two latrines and water taps. Most sewage is collected in open drainage gutters which tend to overflow during the rainy season, flooding adjacent homes. Kolkata has three seasons, the cool dry months from November to February, the hot dry period from March to May, and the monsoon season from June to October. Seventy percent of the people are Hindus, and the rest are predominately Muslims.

This study used the data of a cluster-randomized, double-blind, placebo-controlled trial of a killed oral cholera vaccine [12]. The clusters were dwellings, which were randomly assigned to receive either vaccine or placebo, so that individuals living in the same dwelling (cluster) received the same agent (vaccine or placebo). A dwelling was defined as a hut, a group of huts, or a multistory building with several households using shared water pipes, bathrooms, and latrines as assigned by the Kolkata Municipal Corporation. There were 3933 clusters of which 1966 clusters were assigned to receive vaccine and 1967 clusters were assigned to receive placebo. Residents were eligible to receive a study intervention if they were aged one year or older and were not pregnant.

Nine clinics were established in the community to undertake diarrhea surveillance in addition to the two referral hospitals (Infectious Diseases Hospital and B.C. Roy Children’s Hospital). Private medical practitioners were encouraged to refer patients with diarrhea to the clinics. Patients from the study area were identified by use of household identification cards and a computerized database. Study physicians recorded pertinent clinical details on a structured clinical data form. Rectal swabs were obtained from all patients presenting with history of loose stools and transported in Cary-Blair media to a laboratory at the National Institute of Cholera and Enteric Diseases (NICED) within 8 h of specimen collection. At the laboratory, rectal swabs were examined for *V*. *parahaemolyticus* and *V. cholerae* by use of conventional methods [13].

The disease surveillance data of four years from January 1, 2007 to December 31, 2010 were used in this analysis. A diarrheal visit was defined as a visit by a patient who had, in the 24 h before presentation, three or more loose or liquid stools; or at least one loose or liquid stool with blood; or, if one to two or an indeterminate number of loose or liquid stools were reported, at least some evidence of dehydration, according to WHO criteria [14]. The onset of a diarrheal visit was the day on which the patient first experienced loose or liquid stools. Diarrheal visits for which the date of onset was less than or equal to 7 days from the date of discharge for the previous visit were grouped into the same diarrheal episode. A cholera episode was one in which *V. cholerae* O1 or O139 was isolated from a fecal specimen during any component diarrheal visit; an episode of *V. parahaemolyticus* diarrhea was one in which *V. parahaemolyticus* was isolated.

### Spatial clusters

To detect potential geographic areas of high risk for the diseases, the spatial scan test has been widely used in recent times [15-18]. The spatial scan test is also suitable for uneven geographic distribution of cases and population density [19]. We used the spatial scan test implemented through SaTScan® [16] to identify unique non-random spatial clusters that are higher risk for cholera and for *V. parahaemolyticus*. We assumed that the incidences of two diseases followed a Poisson distribution. Under the null hypothesis, the incidence of disease in a particular location is proportional to the number of residents in that location [19]. Using SaTScan®, we estimated the probability that the frequency of disease at each peak surpasses that expected by chance. We set the space limitations to 50% population at risk, which allowed us to scan for both large and small clusters of disease risk. We took into account the observed number of cases inside and outside the circle when calculating the highest likelihood for each circle. This circle was the most probable cluster and had a rate that was the least likely to happen by chance alone. The statistical significance of possible clusters was calculated using 999 Monte Carlo simulations [20]. Purely spatial analysis was performed using circular windows. The output from SaTScan® was imported into ArcGIS (Version 9.2, California, USA) to map significant (*p<*0.01) clusters of higher risk.

### Statistical analysis

This is an observational population based study in which the population was classified in clusters of residential dwelling units. The study participants were geographically identified based on their residence, and this spatial component was included in the study. Several demographic and socioeconomic variables that were thought to be independently associated with the risk for the diseases were evaluated in the study. However, this is an exploratory analysis of a secondary data source. To avoid distortions due to the impact of the cholera vaccination on the outcomes, we limited our analysis to residential dwellings assigned to placebo. Comparison of individual characteristics was performed using generalized estimating equations (GEE) with logit link function adjusting for the design effect of the clusters used to allocated subjects to the two agents in the randomized trial [21]. To analyze the risk factors of *V. parahaemolyticus* diarrhea or cholera, we used survival analyses of the time to the first episode of the disease, censoring the follow-up of individuals who died or migrated out [22]. We fitted unadjusted and covariate adjusted Cox proportional hazard regression models verifying first that the proportionality assumption was satisfied for all independent variables [23-25]. In both covariate-unadjusted and covariate-adjusted analyses of the risk for *V. parahaemolyticus* diarrhea or cholera, we accounted for the design effect induced by cluster randomization by use of robust sandwich variance estimates [25]. These estimates enabled inferences about risk of the disease at the individual level, adjusting for the design effect. Final adjusted risk estimates were obtained from the model significant at p<0·05 in a forward selection algorithm.

### Ethics

The study received approval from the Health Ministry Screening Committee of the Government of India, Scientific Advisory Committee and Institutional Ethics Committee of NICED, Institutional Review board of International Vaccine Institute and also from the Secretariat Committee for Research Involving Human Subjects, World Health Organization Geneva, Switzerland.

## Results

A total of 54,519 individuals from the placebo clusters (who had not been assigned to receive the oral cholera vaccine) and still resided in the study as of January 1, 2007 were included in the data analysis. Out of these persons, 3,345 (6%) were dropped (died or migrated out) before completing one year of follow-up; 6,334 (12%) were dropped before completing two years of follow-up; 9109 (17%) were dropped before completing three years of follow-up and 12,175 (22%) were dropped before completing four years of follow-up. In total 18,087 diarrhea episodes were observed in this population during the four years of follow-up. All episodes of cholera were due to *V. cholerae* O1, El Tor biotype. The incidence of cholera (1.30/1000/year) was higher than that of *V. parahaemolyticus* diarrhoea (0.63/1000/year), and the difference in incidences between these two *Vibrio* species is statistically significant (p-value <.001). Cholera incidence was higher in younger age groups, but the incidence of *V. parahaemolyticus* diarrhea showed slight increase with age (Table 1). Most of the clinical symptoms of the etiologies of diarrhea were similar (Table 2), but abdominal pain was more common in *V. parahaemolyticus* diarrhea (p-value<.001), and severe dehydration was more common in cholera (p-value<.01). Two out of 137 patients infected by *V. parahaemolyticus* had blood in stool.

**Table 1:** **Number of episodes (incidence rate/1000/year) of *V. parahaemolyticus *diarrhea and cholera in the study area during 2007–2010**

**All group**	**Population at Jan 1, 2007**	***V. parahaemolyticus *Diarrhea No. of episodes (incidence rate/1000/year)**	**Cholera No. of episodes (incidence rate/1000/year)**
<5 years	3,470	7 (0.50)	55 (3.96)
5-14 years	9,873	26 (0.66)	80 (2.03)
15 years+	41,176	104 (0.63)	149 (0.90)
All ages	54,519	137 (0.63)	284 (1.30)

**Table 2:** **Clinical symptoms of *V. parahaemolyticus *diarrhea and cholera in the study area, 2007–2010. No (%)**

**Clinical symptom**	***V. parahaemolyticus *diarrhea (n=137)**	**Cholera (n=284)**	p-value*
Vomiting	53 (38.69)	124 (43.66)	0.333
Nausea	72 (52.55)	122 (42.96)	0.064
Blood in stool	2 (1.46)	0 (0.00)	0.105
Watery stool	106 (77.37)	236 (83.10)	0.159
Drowsy	4 (2.92)	13 (4.58)	0.418
Seizure	0 (0.00)	0 (0.00)	-
Abnormal mental state	0 (0.00)	2 (0.70)	1.000
Abdominal distention	21 (15.33)	25 (8.80)	0.044
Abdominal pain	94 (68.61)	116 (40.85)	<.001
Fever	16 (11.68)	18 (6.34)	0.060
Severe dehydration	18 (13.14)	82 (28.87)	<.001

*The p-values were derived from Chi-square or Fisher’s Exact test.

**Figure 1:**
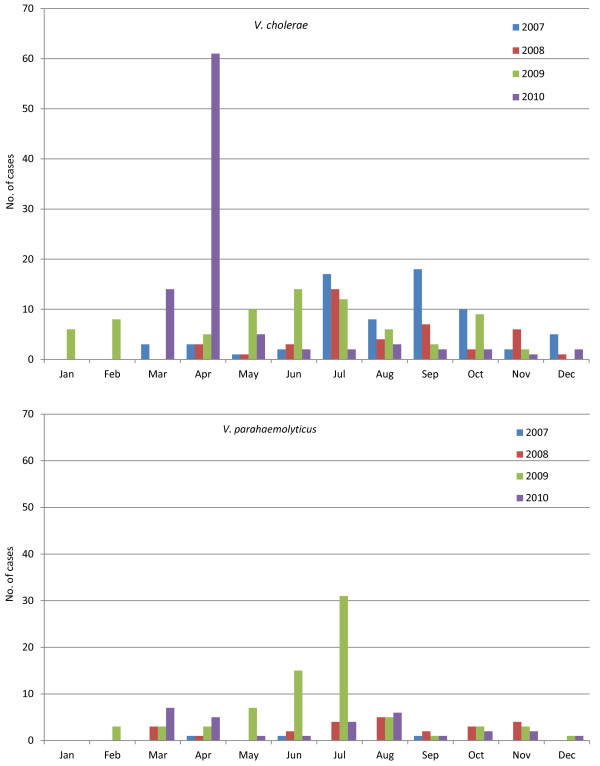
**Number of cases of *V. parahaemolyticus *and *V. cholerae *by month during the study period (2007–2010).**

**Figure 2:**
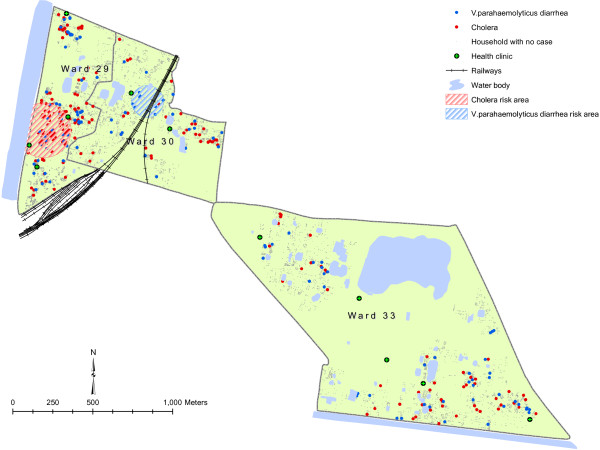
**Spatial distribution of the cases of *V. parahaemolyticus *diarrhea and cholera and the high risk areas of cholera in the study area, 2007–2010.**

Apart from the cholera outbreak in April 2004, the peak season for both the cases of cholera and *V. parahaemolyticus* diarrhea started in the month of July. The peak for cholera continued for a longer period (three months), whereas the peak for the *V. parahaemolyticus* infection lasted only one month (Figure 1). The spatial patterns of the cases for V *parahaemolyticus* and cholera are shown in Figure 2. A significant geographic cluster of *V. parahaemolyticus* diarrhea was detected in a part of ward 30, and a cluster at significantly higher risk for cholera was observed in a part of ward 29.

Table 3 shows socio-demographic characteristics of the study population for cases versus non-cases of *V. parahaemolyticus* diarrhea and cholera separately. There was one subject who experienced the two infections on different occasions and thereby appeared as a case in each analysis*.* The results of the multivariable models for *V. parahaemolyticus* diarrhea and cholera that used the data of the Table 3 in a forward selection algorithm are presented in Table 4 and Table 5, respectively. Hindus and persons living in households not using boiled or filtered water were at greater risk for *V. parahaemolyticus* diarrhea. On the other hand, several indicators of lower socioeconomic status, including not having a household economic contributor with a stable occupation and not having any luxury item were associated with the risk of cholera. Younger subjects and persons living closer to a project healthcare facility were also more likely to be diagnosed with cholera. However, proximity to a project healthcare facility was not associated with higher incidence of *V. parahaemolyticus* diarrhea.

**Table 3:** Socio demographic characteristics of the diarrhea cases infected by the two vibrio species in the study area, 2007–2010

**Variables**	***V. parahaemolyticus***			***V. Cholerae***		
	**Case (n=136)**	**NonCase (n=54,383)**	p-value*	**Case (n=284)**	**NonCase (n=54,235)**	p-value*
Mean (SD) age (years)	29.49 (18.23)	29.80 (18.32)	0.80	20.60 (19.54)	29.85 (18.30)	<.01
No. (%) of children aged less than 5 years	14 (10.29)	3,456 (6.35)	0.06	79 (27.82)	3,391 (6.25)	<.01
No. (%) of males	68 (50.00)	29,095 (53.50)	0.48	152 (53.52)	29,011 (53.49)	0.91
No. (%) of Hindus	100 (73.53)	34,621 (63.66)	0.02	126 (44.37)	34,595 (63.79)	<.01
No. (%) of individuals living in a household with literate household head	99 (72.79)	39,511 (72.65)	0.94	176 (61.97)	39,434 (72.71)	<.01
No. (%) of individuals living in a household with household head having more than 5 years of schooling	75 (55.15)	30,136 (55.41)	0.85	131 (46.13)	30,080 (55.46)	0.07
No. (%) of individuals living in a household with important economic contributor having stable occupation^1)^	27 (19.85)	12,444 (22.88)	0.45	38 (13.38)	12,433 (22.92)	<.01
No. (%) of individuals living in a household using safe toilet^2)^	12 (9.16)	5,002 (9.34)	0.94	8 (3.09)	5,006 (9.37)	0.02
No. (%) of individuals living in a household using safe water source (tap at household level)	14 (10.29)	8,825 (16.23)	0.09	24 (8.45)	8,815 (16.25)	<.01
No. (%) of individuals living in a household using safe water source (tap or hand pump at household level)	14 (10.29)	8,898 (16.36)	0.09	24 (8.45)	8,888 (16.39)	<.01
No. (%) of individuals living in a household using boiled or filtered water	6 (4.41)	6,144 (11.30)	0.01	15 (5.28)	6,135 (11.31)	0.03
No. (%) of individuals living in a household always wash hands with soap and water after defecation	91 (66.91)	37,880 (69.65)	0.56	167 (58.80)	37,804 (69.70)	<.01
No. (%) of individuals living in a household having specific place for waste disposal	133 (97.79)	52,855 (97.25)	0.80	274 (96.48)	52,714 (97.26)	0.28
No. (%) of individuals living in their own house	46 (33.82)	18,654 (34.31)	0.96	62 (21.83)	18,638 (34.37)	<.01
No. (%) of individuals living in a household owning refrigerator	25 (18.38)	11,472 (21.09)	0.48	27 (9.51)	11,470 (21.15)	<.01
No. (%) of individuals living in a household owning motorbike	8 (5.88)	3,943 (7.25)	0.52	11 (3.87)	3,940 (7.26)	0.14
No. (%) of individuals living in a household owning telephone	23 (16.91)	11,644 (21.41)	0.20	24 (8.45)	11,643 (21.47)	<.01
No. (%) of individuals living in a household owning television	94 (69.12)	39,674 (72.95)	0.31	177 (62.32)	39,591 (73.00)	<.01
No. (%) of individuals living in a household owning all luxury items^3)^	4 (2.94)	2,562 (4.71)	0.32	3 (1.06)	2,563 (4.73)	0.01
No. (%) of individuals living in a household owning at least one luxury item^3)^	94 (69.12)	40,221 (73.96)	0.20	180 (63.38)	40,135 (74.00)	<.01
Mean (SD) monthly household expenditure (Indian Rupee)	3155.64 (1462.8)	3822.67 (5753.7)	<.01	3233.93 (1816.5)	3824.10 (5760.5)	<.01
No. (%) of individuals with high monthly household expenditure^4)^	43 (32.33)	24,306 (45.36)	<.01	88 (31.43)	24,261 (45.40)	<.01
Mean (SD) monthly per-capita expenditure^5)^ of house hold (Indian Rupee)	540.79 (273.90)	675.16 (1817.8)	<.01	495.57 (305.13)	675.77 (1820.2)	<.01
No. (%) of individuals having high monthly per-capita expenditure^4)^ of household	49 (36.84)	25,689 (47.94)	0.02	85 (30.36)	25,653 (48.00)	<.01
Mean (SD) Mean (SD) of the household size	6.79 (3.54)	6.90 (3.78)	0.78	7.72 (4.15)	6.89 (3.77)	0.01
Mean (SD) distance (m) from household to the nearest health clinic	173.40 (107.00)	179.91 (103.46)	0.60	145.99 (77.90)	180.07 (103.56)	<.01
No. (%) of individuals living in household longer distance to the nearest health clinic^6)^	61 (44.85)	27,193 (50.00)	0.39	82 (28.87)	27,172 (50.10)	<.01
Mean (SD) distance (m) from household to the nearest water body	106.31 (57.79)	101.71 (57.98)	0.32	117.64 (59.21)	101.64 (57.96)	<.01
No. (%) of individuals living in household longer distance to the nearest water body ^7)^	74 (54.41)	27,184 (49.99)	0.35	170 (59.86)	27,088 (49.95)	0.02
No. (%) of individuals living in Ward 33	46 (33.82)	14,668 (26.97)	0.10	57 (20.07)	14,657 (27.02)	0.05

^*^ The p-values were derived by comparing the differences between the two groups adjusted for cluster effects using GEE with the logit link function.

^1)^ Stable occupation: professional, service, shop owner, or farm owner.

^2)^ Safe toilet: use of own (non-shared) flush toilet.

^3)^ Luxury items: Refrigerator, motorbike, telephone, and/or television.

^4)^ High (household/per-capita household) expenditure: more than the median household/per-capita household expenditure; median derived using the vaccinated population [0: low expenditure; 1: high expenditure].

^5)^ Per-capita monthly expenditure: calculated from total household expenditure divided by the number of active members in the household prior to vaccination (1USD = 45 Indian Rupees).

^6)^ Longer distance to the nearest health clinic: more than the median distance to the nearest health clinic; median derived using the vaccinated population [0: close to the nearest health clinic; 1: long distance to the nearest health clinic].

^7)^ Longer distance to the nearest water body: more than the median distance to the nearest water body; median derived using the vaccinated population [0: close to the nearest water body; 1: long distance to the nearest water body].

**Table 4:** **Predictors of the risk of *V. parahaemolyticus *diarrhea in the study area, 2007–2010**

**Variables**	HR*	**95% CI**	**P-value**
Hindus	1.79	1.21-2.66	0.0037
Individuals living in a household using boiled or filtered water	0.32	0.14-0.72	0.0061

*Hazard ratio for the cited variable, adjusted for all other variables in the table.

**Table 5:** Predictors of the risk of cholera in the study area, 2007–2010

**Variables**	HR*	**95% CI**	**p-value**
Individuals 5 years and above	0.47	0.40-0.55	<.0001
Individuals living in a household with important economic contributor having stable occupation^1)^	0.64	0.42-0.99	0.0449
Individuals living in a household using safe toilet^2)^	0.44	0.16-1.22	0.1139
Individuals living in a household owning at least one luxury item^3)^	0.72	0.54-0.95	0.0196
Longer distance from the household to the nearest health clinic^6)^	0.46	0.33-0.63	<.0001

Note, Please see the footnotes of Table 3 for the citations.

*Hazard ratio for the cited variable, adjusted for all other variables in the table.

## Discussion

This paper reports coexistence of *V. parahaemolyticus* and *V. cholerae* in the slums of Kolkata, India. Literature suggests foods frequently incriminated in *V. parahaemolyticus* infections are raw or inadequately cooked seafood and foods contaminated by seafood materials [26]. However, the transmission and epidemiology of *V. parahaemolyticus* infections in the study area may be different because seafood is never eaten raw and freshwater fish is preferred over seawater fish by the local people.^3^ Contamination by seawater fish at the fish market, and secondary contamination of other foods in the kitchen by *V. parahaemolyticus*-contaminated fish brought from markets as well as cutting and dressing of sea food especially prawn (affecting women more) may be the routes of transmission of the *V. parahaemolyticus* in the study area [27-29].

The clinical symptoms and seasonality for both the diseases are almost identical, thus it is difficult to diagnose the patients without microbiological test of the patients’ fecal specimens. However, some degree of differentiation of these patients may be made by looking for severity of diarrhoea and stomach pain. The incidence of cholera was higher than that of *V. parahaemolyticus* diarrhoea in the study area. The incidence rates reported here are certainly underestimates of the true rates, because the cases were detected only through augmented passive surveillance in project health clinics. The higher risk for cholera for people living closer to the project health facilities also suggests that some patients living far from the project health facilities might not have sought their care for diarrhea from the project health clinics. The proportion of patients with diarrhea in the study area seeking treatment in those clinics was not assessed.

It was interesting to note that the Hindus, the dominant religious group, were at greater risk for *V. parahaemolyticus* diarrhea compared to Muslims*.* The Hindus are relatively affluent in that society in comparison to Muslims. A study in coastal Vietnam [26] showed that more affluent members of the community, assessed by their higher professional status, better living conditions, and possession of luxury objects, were more frequently infected by *V. parahaemolyticus* compared to less affluent individuals. A possible explanation for that finding could be that only the more affluent members of the community can afford to include fish in their diet, which is thought to be the source of infection.

The cluster of significantly higher risk for cholera was observed in a part of Ward 29 where the density of population is very high. A study observed that the density of refuse dumps is an important environmental predictor of cholera [30]. The refuse dump was considered as an index of basic sanitation in that study. It is reasonable to believe that an area with higher density of population may create increased density of refuse dumps, and the resulting breakdown of sanitation can lead to a higher risk for the cholera in the area. Likewise, the cause of clustering of the *V. parahaemolyticus* diarrhea in ward 30 may be explained by the fact that it has predominantly Hindu population and was thereby more prone to *V. parahaemolyticus* diarrhea (as mentioned above). However, further in-depth studies are needed to find the cause of spatial risk for the two organisms in the study area.

## Conclusion

There is not much information on the differences in clinical, epidemiological and spatial characteristics of diarrhea due to *Vibrio Cholerae* and *V parhaemolyticus* from non-coastal areas, and we observed some distinctive risk factors and spatial patterns of risk for diarrhea due to cholera and *V. parahemolyticus* suggesting different modes of transmission of these two pathogens. This information may be helpful for the health policy makers. However, further research is needed to delineate the modes of transmission of the two vibrios.

## Competing interest

The authors declare that they have no competing interest.

## Authors’ contribution

SK, MA, DS, SKB, JC, SKB and GBN contributed to the study design, analysis, and writing of the manuscript. YAU and BM and BB contributed to the data analysis, SK, MA, DS and DP contributed to writing of the manuscript.SKN and BLS helped in laboratory diagnosis of the samples. DS, JC, SKN, SKB and GBN critically reviewed the paper. All authors saw and approved the final version of the manuscript.

## Pre-publication history

The pre-publication history for this paper can be accessed here:

http://www.biomedcentral.com/1471-2458/12/830/prepub
